# Optically Controlled Bias-Free Frequency Reconfigurable Antenna

**DOI:** 10.3390/s25195951

**Published:** 2025-09-24

**Authors:** Karam Mudhafar Younus, Khalil Sayidmarie, Kamel Sultan, Amin Abbosh

**Affiliations:** 1 School of Electrical Engineering and Computer Science, University of Queensland, Brisbane, QLD 4072, Australia; k.sultan@uq.edu.au (K.S.); a.abbosh@uq.edu.au (A.A.); 2 College of Electronics Engineering, Ninevah University, Mosul 41002, Iraq; kh.sayidmarie@uoninevah.edu.iq

**Keywords:** optical switching, bias-free, Light-Dependent Resistor (LDR), reconfigurable antenna, multi-band antenna

## Abstract

A bias-free antenna tuning technique that eliminates conventional DC biasing networks is presented. The tuning mechanism is based on a Light-Dependent Resistor (LDR) embedded within the antenna structure. Optical illumination is used to modulate the LDR’s resistance, thereby altering the antenna’s effective electrical length and enabling tuning of its resonant frequency and operating bands. By removing the need for bias lines, RF chokes, blocking capacitors, and control circuitry, the proposed approach minimizes parasitic effects, losses, biasing energy, and routing complexity. This makes it particularly suitable for compact and energy-constrained platforms, such as Internet of Things (IoT) devices. As proof of concept, an LDR is integrated into a ring monopole antenna, achieving tri-band operation in both high and low resistance states. In the high-resistance (OFF) state, the fabricated prototype operates across 2.1–3.1 GHz, 3.5–4 GHz, and 5–7 GHz. In the low-resistance (ON) state, the LDR bridges the two arcs of the monopole, extending the current path and shifting the lowest band to 1.36–2.35 GHz, with only minor changes to the mid and upper bands. The antenna maintains linear polarization across all bands and switching states, with measured gains reaching up to 5.3 dBi. Owing to its compact, bias-free, and low-cost architecture, the proposed design is well-suited for integration into portable wireless devices, low-power IoT nodes, and rapidly deployable communications systems where electrical biasing is impractical.

## 1. Introduction

Wireless systems face increasing pressure to operate across multiple bands and contexts while minimizing node size, cost, and power consumption. In reconfigurable systems, the tuning technique becomes just as important as what is being tuned. Traditional electrical tuning relies on bias networks (DC lines, RF chokes, blocking capacitors, control ICs) that introduce loss, parasitics, routing challenges, failure points, and significant power consumption. These overheads are especially problematic in compact platforms and battery-powered IoT nodes, where every milliwatt and millimeter count [1,2,3,4].

Rising wireless demands require versatile antennas that adapt across operating conditions [5,6]. Reconfigurable antennas dynamically adjust frequency, pattern, or polarization, reducing hardware duplication and enabling compact systems [5]. In particular, frequency reconfigurability consolidates multi-band operation, lowering complexity and cost while improving performance. Consequently, extensive research has yielded diverse implementations spanning across satellite, MIMO, and cognitive-radio applications [7,8]. Reconfiguration techniques can be broadly classified into four categories: electrical, mechanical, smart-materials-based, and optical [7,9,10]. Among these, electrical-based reconfiguration remains the most prevalent, employing active components such as PIN diodes, varactor diodes, and radio-frequency microelectromechanical system (RF-MEMS) switches [11,12,13]. A common challenge across electrical-based methods is the inherent requirement for bias networks and associated control circuitry [9].

In electrically reconfigured systems, the bias network is not RF-transparent. It couples into RF currents, introduces lumped parasitics, constrains layout, and often requires vias, RF chokes, blocking capacitors, or isolation slots that degrade device performance. For antennas, these effects worsen as the antenna becomes smaller. Also, at millimeter-wave, diode packages plus bias lines measurably alter return loss and patterns unless carefully mitigated; similar trends hold at sub-6 GHz when bias lines share current paths or impose ground discontinuities. Removing the bias network eliminates these compromises at the root [6,7,14].

PIN diodes are widely adopted as switching elements. However, their integration demands extra RF or DC biasing blocks and sufficient forward current to achieve a low ON state. As a reference point, common PIN limiter/switch diodes specify around 1–2 Ω series resistance at 10 mA forward bias, implying control power on the order of 5–20 mW per element, depending on bias voltage and duty-cycle [15]. Varactors, while offering continuous tuning with negligible DC in reverse bias, still require biasing circuits that add loss and complexity. RF-MEMS switches exhibit excellent RF linearity and near-zero static power but typically require tens of volts for electrostatic actuation (and the associated high-voltage driver), raising integration overhead even when average power is low. These technology-level trade-offs motivate alternatives that decouple reconfigurability from electrical biasing altogether [4,11,15]. This is particularly important for energy-limited applications, such as IoT.

Many IoT devices operate for months to years on coin cells or harvested energy, with typical standard discharge currents around 0.4 mA and maximum continuous currents near 3 mA for a CR2032 cell. In such devices that require reconfigurable antennas, there is little headroom for multi-milliamp PIN bias rails or high-voltage MEMS drivers [16]. Eliminating DC bias circuits removes both their static quiescent draw (regulators, DACs, GPIO rails) and their dynamic actuation energy, aligning antenna tuning with the ultra-low-power regime [16]. In contrast, mechanical reconfiguration achieves adaptability by physically altering the antenna structure [17,18]. This method avoids the integration of active elements and biasing systems, simplifying the electrical design, but often at the cost of limited performance flexibility and slower actuation. Importantly, this approach is not suitable for IoT devices that might be planted in remote locations.

Beyond conventional techniques, smart-materials-based reconfiguration is an emerging area [19,20]. While still facing challenges in reliability and efficiency, several notable designs have been reported [21,22]. This approach often manipulates the electromagnetic properties of the substrate, for example, pumping fluid into hollow structures to alter effective permittivity/permeability [23], leveraging materials such as graphene [24], or using liquid metal [2].

Unlike the aforementioned techniques, optical approaches can place the tuning element directly in the RF path without any DC lines on the RF board. Prior work has demonstrated optically controlled reconfigurable elements (e.g., photoconductive switches, photodetectors) to toggle radiating structures, achieving pattern or frequency agility with no on-board DC bias network [25,26]. Building on this thread, we employ a Light-Dependent Resistor (LDR) as a compact, low-cost photoconductive element whose resistance collapses under illumination (retuning or switching the antenna) and reverts to a high value in darkness. The absence of bias lines simplifies the RF layout, removes RF chokes, and preserves radiator current paths, particularly valuable in compact reconfigurable antennas where bias routing would otherwise intersect strong surface currents. When ambient light is undesirable, a guided or shrouded optical illumination path provides deterministic control without reintroducing RF or DC routing.

Table 1 compares reconfiguration mechanisms on bias needs, power, speed, and layout impact. In particular, the LDR is unique in simultaneously eliminating RF-board bias lines and RF-board quiescent power while imposing minimal layout overhead at the cost of millisecond-range switching that remains adequate for band selection in low-energy nodes.

This work proposes a bias-free, optically controlled reconfigurability concept that removes DC bias circuitry, simplifies integration, reduces parasitic loading, and aligns antenna tuning with ultra-low-power system budgets. Conceptually, the approach is geometry-agnostic and can be applied to any antenna topology. The specific monopole adopted in this work is used to validate the concept rather than the contribution itself. The concept is validated using full-wave simulations and experiments.

## 2. Antenna Design, Reconfiguration Mechanism, and Parametric Analysis

### 2.1. Antenna Structure and Reconfigurable Operation

**Figure 1:**
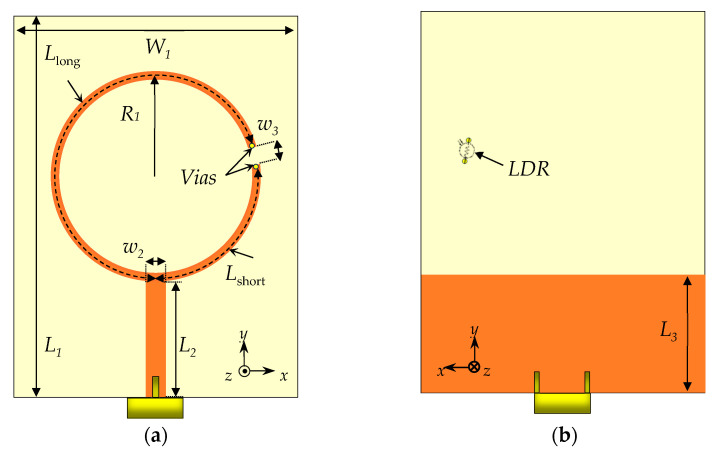
Schematic of the proposed antenna. (**a**) Top view and (**b**) bottom view. Dimensions (mm): *L*_1_ = 56.0, *L*_2_ = 17.0, *L*_3_ = 17.0, *W*_1_ = 42.0, *w*_2_ = 2.9, *w*_3_ = 2.9, *R*_1_ = 16.8, *L*_short_ = 27.0, *L*_long_ = 76.3.

To demonstrate the proposed concept, a basic ring monopole antenna printed on the top layer of the substrate is utilized. This antenna, shown in Figure 1, includes a slot at a specific location to control its operating modes. A 50 Ω microstrip feedline, also on the top layer, excites the monopole. The bottom layer hosts a partial ground plane covering the lower portion of the substrate, with the monopole positioned entirely above the ground plane. An LDR is placed at the slot between the ends of the two arcs forming the monopole, enabling optical switching between isolated and connected states. Specifically, the LDR is mounted on the backside of the substrate, and its two terminals are connected to the ends of the slot through vertical vias. Each via extends through the 1.6 mm substrate, and the LDR itself has a body length of approximately 3.2 mm. As a result, the LDR introduces an additional conductive path (*L*_bridge_) of approximately 6.4 mm when the two arcs are electrically connected in the ON state.

The arc lengths are calculated using *L* = *r·θ*, where *r* is the average radius and *θ* is the central angle in radians. The monopole width is 1 mm. The antenna is built using the FR-4 substrate with relative permittivity εr = 4.3 and loss tangent tanδ = 0.025.

When the LDR is in the ON state (low resistance), the two arcs and the LDR bridge form a closed conducting path of length:

*L*_ring_* = L*_short_* + L*_long_* + L*_bridge_. (1)

A uniform loop resonates when the total phase around the path equals 2π*n* [27,28]:

β*L*_ring_ = 2π*n*, (2)

with

(3) $$\beta =\frac{2\pi }{\lambda_{g}}=(\frac{2\pi f}{c})\;\sqrt{\varepsilon_{eff}},$$

where *β* is the phase constant of the guided mode along the ring path, *λ_g_* is the guided wavelength, *c* is the speed of light, and *n* is the mode number. Here, *ε_eff_* is the effective dielectric constant [29]:

(4) $$\varepsilon_{eff}=\frac{\varepsilon_{r}+1}{2},$$

where *ε_r_* is the substrate’s relative permittivity. Substituting (3) into (2) results in

(5) $$f_{n}=\frac{nc}{L_{ring}\sqrt{\varepsilon_{eff}}}$$

**Figure 2:**
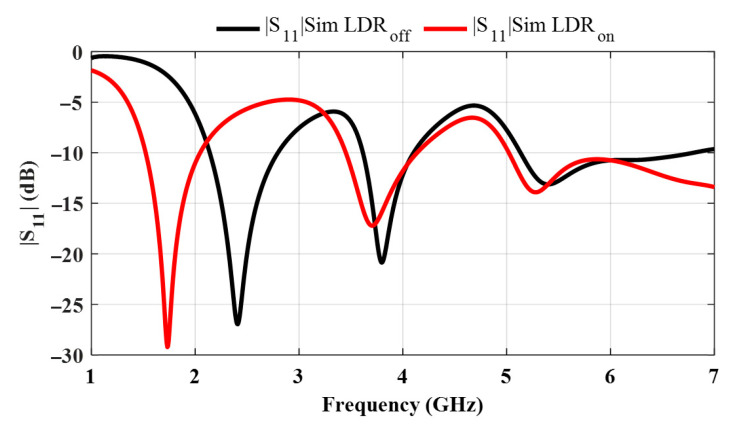
Simulated reflection coefficient variation with frequency when the LDR is in the ON (illuminated) and in the OFF (not illuminated) states.

Consequently, in the ON state, the closed ring supports the simulated bands of 1.52–2.05 GHz, 3.45–4.13 GHz, and 5.02 GHz to beyond 7.00 GHz using the |S_11_| ≤ −10 dB criterion. The operation in these bands represents Mode (1), Mode (2), and Mode (3), respectively, where the ring circumference equals *λ*_e_, 2*λ*_e_, and 3 *λ*_e_, as shown in Figure 2.

In the OFF state (high resistance), the feed excites two curved arc resonators in parallel (*L*_short_ and *L*_long_). The short arc supports the band between 5.13 and 6.80 GHz, while the long arc supports 2.14–2.80 GHz. Since the two open branches are fed in parallel, a third resonance appears where their shunt susceptances cancel (standard two-stub behavior) [28], producing the mid-band observed between 3.61 and 4.10 GHz (see Figure 2).

Table 2 summarizes the calculated and simulated resonance frequencies of the antenna, demonstrating close agreement between analytical estimates and full-wave simulations.

### 2.2. LDR Integration and Electrical Switching

The LDR used in this design is the NSL-6112, a CdS-based photoresistor with a ceramic two-lead package, offering reliable visible light sensitivity in the 400–700 nm range. Its electrical resistance varies significantly depending on light intensity: in dark conditions, the resistance is 1.1 to 2 kΩ at (324-21 lux), and ≥1.3 MΩ at (≤5 lux) with the long response of 3 s as a fully dark cell relaxes toward very high resistance, effectively acting as an open circuit [30]. However, under illumination (e.g., 1076 lux), it drops to around 170 Ω and can be further reduced to below 25 Ω by increasing the illumination to approximately 5000 lux, providing a low-resistance conductive path between the two crescent arc arms. In addition to these values, two aspects are key to practical implementation: switching speed and temperature dependence [31]. Under bright, focused LED illumination (5000 lux), the ON transition is within the millisecond range, whereas at very low illuminance, the turn-off can be slower [31,32]. At a fixed illuminance, CdS-LDR resistance decreases with ambient temperature (negative temperature coefficient), and typical parts operate within the range −60 °C to +75 °C [30]. All measurements in this work were performed near room temperature. This optical switching mechanism is used to control the reconfiguration state of the antenna. The LDR is mounted on the backside of the FR-4 substrate, with each lead connected to the terminal point of the crescent arcs through vias. Electrically, the LDR can be modeled using a variable resistor *R_LDR_* controlled by illumination, in parallel with a small parasitic capacitance *C_p_* (from the ceramic package) and a series parasitic inductance *L_p_* (due to the wire leads). Typical literature values for the dark-state capacitance are in the order of 3 to 4 pF, which can increase under illumination (device-dependent), while the lead inductance is small (sub-nH to a few nH) [25,30]. These values were included in the electromagnetic simulations to ensure accurate representation of the switching element’s high-frequency behavior. This passive, light-controlled nature allows bias-free, real-time switching between isolated and connected arc states, enabling tri-band operation of the antenna with no additional active circuitry. The compact size and low-power characteristics of the NSL-6112 make it a highly suitable choice for this type of low-profile, optically controlled reconfigurable antenna. While CdS photoresistors might age under sustained illumination, leading to a gradual increase in ON states over long periods, stability improves with moderate illuminance, controlled temperature, and good light isolation [31]. The implementation uses a compact green LED near the CdS sensitivity peak, a matte-black micro-shroud to block ambient light and UV, and modest drive levels to limit heating [31]. For applications that need tighter long-term stability, hermetic (TO-can) CdS parts or silicon photoresistive sensors are alternatives, noting they have different responsivity and might need retuning of illuminance [31,32].

## 3. Simulated and Measured Results

**Figure 3:**
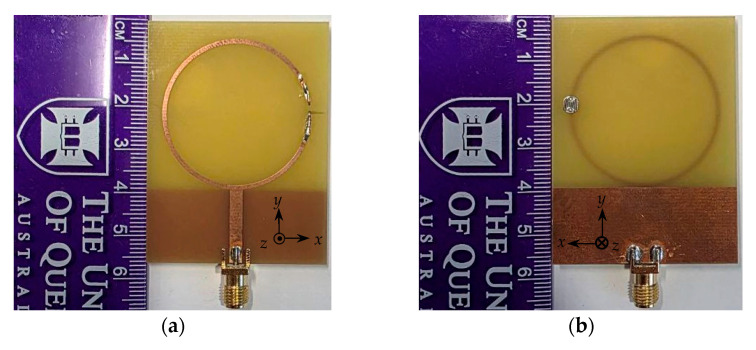
Photographs of the fabricated prototype. (**a**) Front view and (**b**) back view.

The proposed antenna was simulated in CST Microwave Studio (time-domain solver). The SMA footprint and feed transition were modeled explicitly, with the port reference plane at the pad edge. The LDR between the crescent-arc terminals was modeled as an illumination-dependent resistor R_LDR_ in parallel with Cp = 3.5 pF and in series with Lp = 0.6 nH. Two operating cases were evaluated using measured resistances: OFF (R_LDR_ ≈ 1.1 kΩ, indoor light ≈ 324 lux) and ON (R_LDR_ ≈ 25 Ω, focused white LED ≈ 5000 lux at 20 cm). A prototype (Figure 3) was characterized in an anechoic chamber under the same illumination conditions used in simulation. The LDR resistances measured in the situation matched the simulation inputs (≈1.1 kΩ in the OFF state and ≈ 25 Ω in the ON state), enabling direct comparison of simulated and measured results without re-parameterization.

**Figure 4:**
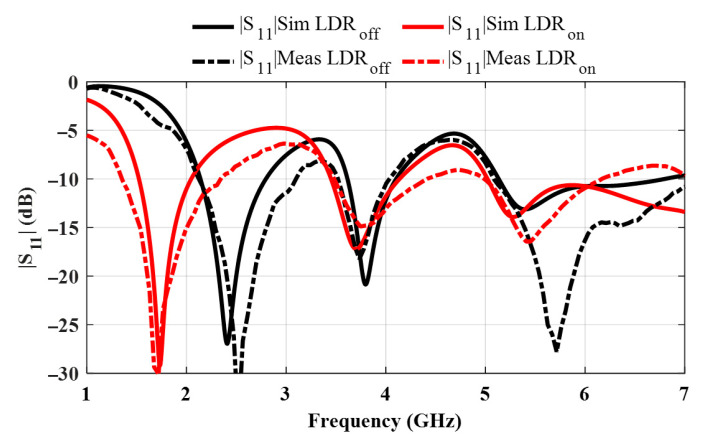
Simulated and measured reflection coefficients of the antenna for the ON and OFF states of the LDR.

The simulated and measured reflection coefficients are analyzed for both ON and OFF states of the antenna, corresponding to the closed and open conditions of the LDR, respectively (see Figure 4). In this paper, the operating bands are determined based on the |S_11_| ≤ −10 dB criterion for both simulation and measurement. In the OFF state, where the LDR behaves as an open circuit and the two arcs are disconnected, the simulation results reveal three distinct operating bands: 2.14–2.80 GHz, 3.61–4.10 GHz, and 5.13–6.80 GHz. The measured results for the OFF state are in close agreement, showing slightly wider or shifted bands of 2.11–3.1 GHz, 3.5–4 GHz, and 5.08 GHz to beyond 7 GHz, indicating a generally wider and more extended bandwidth, especially at higher frequencies, likely due to additional parasitic coupling and fabrication tolerances.

In the ON state, the LDR forms a conductive bridge between the arcs, effectively extending the total current path. This results in a downward frequency shift in the lower operating band, as observed in the simulated results of 1.52–2.05 GHz, 3.45–4.13 GHz, and 5.02 GHz to beyond 7 GHz. The measured ON state data also reflects this behavior, with operational bands at 1.36–2.35 GHz, 3.47–4.42 GHz, and 5.00–6.17 GHz. Overall, the measurement results are in good agreement with the simulations, validating the reconfigurability of the antenna structure via optical switching and confirming the effectiveness of the LDR in enabling tunable dual- and tri-band behavior.

**Figure 5:**
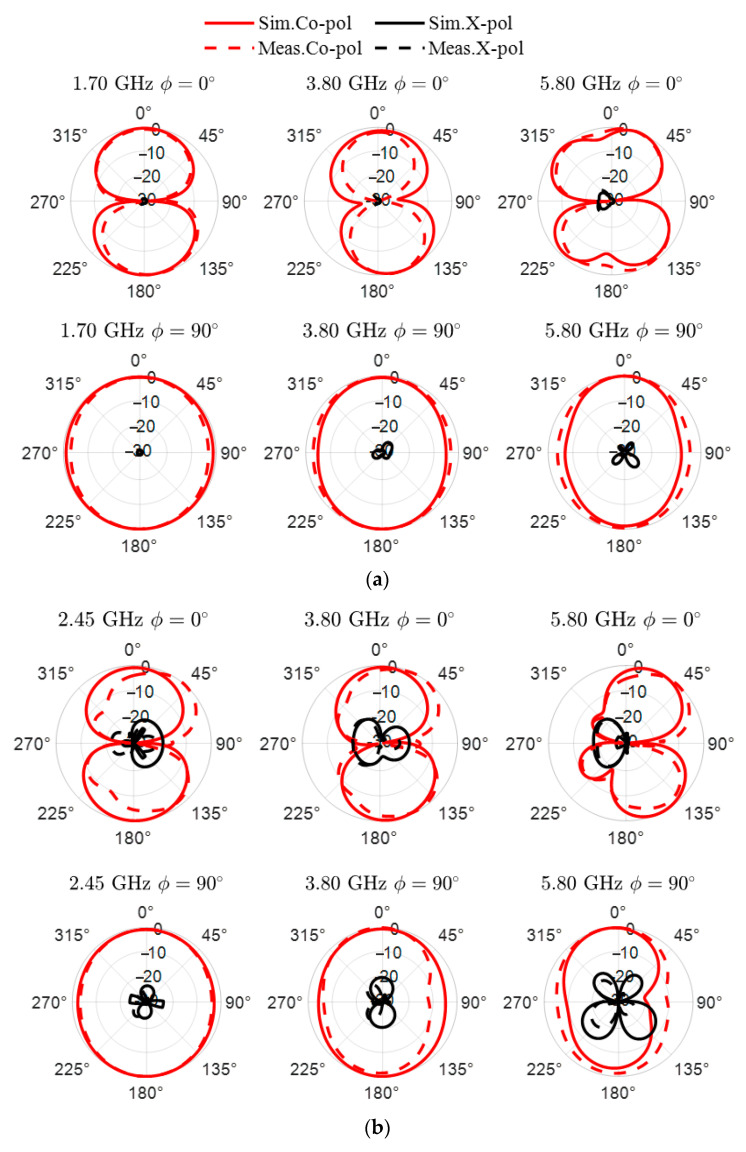
Simulated and measured normalized radiation patterns of the proposed antenna. (**a**) Patterns at 1.70, 3.80, and 5.80 GHz for *xz* and *yz* planes when LDR is in ON state; and (**b**) patterns at 2.45, 3.80, and 5.80 GHz for *xz* and *yz* planes when LDR is in OFF state.

The antenna’s radiation characteristics are evaluated through both simulation and measurement for the ON and OFF states of the LDR, and the obtained results are shown in Figure 5. The measured radiation patterns are in good agreement with the simulated ones, confirming the consistency of the antenna’s directional behavior and polarization properties. In all operating bands and both switching states, the antenna maintains linear polarization, as evidenced by the co- and cross-polarization responses. Cross-polarization is seen as being relatively larger for the upper band, where the antenna length is a multiple of the fundamental resonance length. The radiation patterns exhibit stable main lobes and suppressed cross-polarization, supporting the effectiveness of the proposed reconfigurable structure.

**Figure 6:**
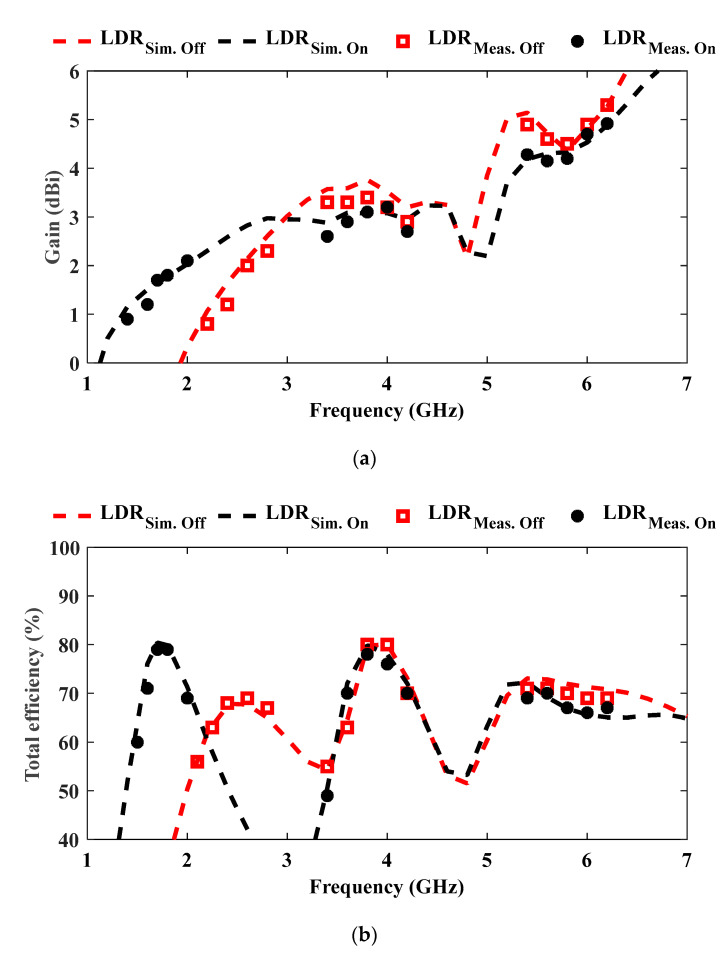
Simulated and measured performance. (**a**) Realized gain across all operating bands for LDR ON and OFF states, and (**b**) total radiation efficiency across all operating bands for LDR ON and OFF states.

The measured gain in the OFF state ranges from 0.8 dBi to 5.3 dBi, with values around 2–2.3 dBi in the lower band, 3.3–3.4 dBi in the mid-band, and up to 5.3 dBi in the upper band. This is attributed to the increased antenna size in terms of the wavelength as one goes to the higher frequency band. In the ON state, the gain lies between 0.9 dBi and 4.92 dBi, typically ranging from 0.9 to 2.1 dBi in the lower band, 2.6–3.2 dBi in the mid-band, and exceeding 4.2 dBi in the high-frequency region, as shown in Figure 6a. It is seen that as the antenna is switched from the ON state to the OFF state, the gain at 1.7 GHz frequency drops by more than 2 dB, while the gain at 2.45 GHz increases by about 2 dB. This finding follows the same trend consistent with the reflection coefficient shown in Figure 4. The antenna at the switched-off band is characterized by a higher reflection coefficient, as well as lower gain.

The measured radiation efficiency remains high across all bands: between 56% and 80% in the OFF state and 60% to 79% in the ON state, as shown in Figure 6b.

## 4. Comparison

Table 3 benchmarks the proposed optically controlled, bias-free reconfiguration against VO_2_, PIN-diode, varactor, liquid-dielectric, and AMC-assisted methods. Beyond bands, gain, and size, it reports integration overhead via five metrics: presence of RF-board DC bias lines, required control drive (current/voltage), RF-board quiescent power, switching speed, and layout intrusion (vias, chokes, bias routing). The LDR approach uniquely requires no RF-board biasing and draws zero RF-board quiescent power while keeping layout intrusion low; the trade-off is millisecond-scale switching, appropriate for band/mode selection in low-power and IoT use cases.

In many deployments, ambient light is sufficient to drive the LDR to the ON state, so no LED is needed, and thus, the RF board draws 0 mW quiescent power. When ambient light is low, a small LED can provide the required illuminance. For the 5000 lux ON condition, illuminating a 2–4 mm diameter spot corresponds to about 0.016–0.063 lumens of light. With a conservative 150 lm/W LED, this translates to roughly 0.10–0.42 mW of electrical power [31,32]. Even if we assume excessive optical and driver losses, the required electrical power stays in the range of 0.2–0.9 mW. By contrast, PIN-diode tuning commonly uses around 1–10 mW per diode from forward bias. The optical approach therefore avoids on-board bias rails and keeps the RF board at 0 mW when relying on ambient light.

Beyond the optical LDR method, two common reconfigurable techniques that avoid on-board DC biasing are mechanical reconfiguration and fluidic/liquid–metal tuning [2,6,18,23]. Mechanical techniques utilizing sliding or rotating elements are entirely bias-free at the RF board, offer linearity, and introduce very low RF loss. However, they add bulky parts, rely on moving parts, and typically exhibit millisecond to second range switching, which creates integration challenges in compact nodes. Fluidic/liquid–metal techniques are also bias-free at the RF board and can provide large, continuous geometry changes with wide tuning. However, they usually require pumps, valves, or other external actuation, have system-dependent millisecond- to second-range switching, and raise concerns about leakage, material handling, and packaging complexity [2,23]. By contrast, the optical approach used here keeps the RF board at 0 mW quiescent power, introduces no pumps or moving parts, preserves current paths by avoiding bias lines, and achieves millisecond-range ON transitions under focused illumination. The CdS LDRs switch in the millisecond range under bright illumination because carrier generation/recombination in the photoconductor is fast, and vendor datasheets specify ~35–100 ms rise and ~5–20 ms fall at ~10 lux, with faster response at higher illuminance [31].

The proposed antenna offers two optically switchable states that both support tri-band operation, with the low band reconfigured between 2.45 GHz and 1.7 GHz, while the mid and high bands remain centered at 3.8 GHz and 5.8 GHz. Regarding the number of switching elements and bias complexity, the proposed design is notably simple: it uses a single LDR and thus avoids RF bias networks and multiple DC lines typical of PIN-based approaches. By contrast, comparable dual-band antennas often require multiple PIN diodes (e.g., [33,34,35,36,37]) or a varactor network as in [38], which increases routing complexity and potential insertion loss. Despite the minimal hardware, the proposed antenna achieves tri-band coverage with wide fractional bandwidths, whereas most compared reconfigurable designs are dual-band only. On bandwidth, the proposed low-band fractional bandwidth exceeds that of [33,36,37,38] and is competitive with the wider responses reported in [34]. While [35] reports an ultra-wide low band, it remains dual-band and still requires multiple PIN diodes, whereas the proposed design provides tri-band operation with only one optical element. Regarding gain, the proposed antenna is higher than the VO_2_-based design in [39], the liquid-dielectric design in [40], the varactor-based design in [38], and several PIN-based designs in [34,35], and it is competitive with the better-performing PIN/AMC examples in [33,36,37]. Thus, the proposed solution achieves mid-to-high gain without resorting to large arrays, AMC surfaces, or numerous diode switches. From a form-factor perspective, the proposed footprint yields an area of 0.048 λ_1_^2^, which is smaller than typical PIN-based designs such as those in [33,34,37] and varactor-based designs in [38]. Overall, the antenna is compact and low-profile while preserving efficient multiband behavior. In summary, when considering switch count, band count, bandwidth, gain, and size/profile simultaneously, the proposed LDR-based reconfigurable monopole compares favorably with the literature and offers a simpler, more integrable path to practical multiband reconfiguration.

**Table 3:** Comparison of the proposed LDR-based reconfigurable monopole antenna with reported designs in the literature.

Ref.	Reconfiguration Method (No of Devices)	RF-Board DC Bias Lines?	Control Drive (typ.)	RF-Board Quiescent Power *	Switching Speed	Layout Intrusion	Resonant Frequency	Relative BW (%)	Max Gain (dBi)	No. of Switch States	Dimensions (λ_l_)
[39]	VO_2_	Yes	Thermal/drive current	mW–100 s mW	ms–s	High	4.12–7.1	5/3	1.73	2	0.28 × 0.09 × 0.007
[33]	PIN diodes (4)	Yes	~10 mA forward	mW per element	ns–µs	High	2.45–3.5	12/6.5	6.8	2	0.8 × 0.8 × 0.02
[34]	PIN diodes (4)	Yes	~mA forward	mW	ns–µs	High	3.5–5.5	33.7/19.8	4.2	2	0.583 × 0.583 × 0.018
[35]	PIN diodes (2)	Yes	~mA forward	mW	ns–µs	Med–High	3.16, 5.6–13.7	104.5/20.8	3.8	2	0.21 × 0.31 × 0.017
[36]	PIN diodes (12)	Yes	~mA forward	mW	ns–µs	High	2.4–5.8	0.7/1.7	6.7	2	0.42 × 0.42 × 0.01
[37]	AMC + PIN diodes (4)	Yes	~mA forward	mW	ns–µs	High	3.5–4.6	27.1/25	8.14	2	0.78 × 0.78 × 0.1
[38]	Varactor (1)	Yes	2–20 V reverse	~0 (device) + driver	ns–µs	Med	4.13–4.50	1.5/2.2	4	2	0.29 × 0.29 × 0.02
[40]	Liquid dielectrics	Often No †	Pumps/valves	System-dependent	ms–s	High	1.29–2.51	13.94/13.9	4.7	2	0.19 × 0.33 × 0.003
[41]	PIN diode (1)	Yes	~mA forward	mW	ns–µs	Med	6.8–15.5	NA	3.8	2	0.68 × 0.363 × 0.008
This work	LDR (1)	No	Optical illumination	0 (on RF board)	ms	Low	OFF state		5.3	2	0.254 × 0.19 × 0.0073
							(2.45/ 3.8/ 5.8)	38.0/13.3/31.8)			
							ON state				
							(1.7/ 3.8/ 5.8)	(53.3/24.1/20.9			

* “RF-board quiescent power” refers to power drawn by components physically on the RF substrate. Optical control keeps the RF board at 0 quiescent power; illumination is off-board. † “Often No” indicates many liquid/fluidic approaches route actuation off the RF board that require external pumps/valves and fluid channels, which increases integration complexity. λ_l_ is the free-space wavelength at the lowest operating frequency.

## 5. Conclusions

This work introduces a bias-free antenna tuning concept that eliminates on-board DC bias networks by optically controlling an LDR embedded in the RF path. Removing bias lines, RF chokes, blocking capacitors, and control ICs reduces parasitics and routing constraints, while crucially bringing the RF-board quiescent power to zero. The optical stimulus also provides isolation from electrical and electromagnetic interference between the controller and the RF front end. The concept is validated by integrating an LDR into an antenna, demonstrating that illumination modulates the LDR resistance in two states, ON and OFF, thereby adjusting the effective electrical path length and enabling frequency reconfigurability without on-board biasing. Full-wave simulations and measurements show consistent results across reflection response, radiation pattern, gain, and efficiency, confirming that optical reconfiguration can deliver multi-band agility while maintaining simple layouts. Because the mechanism controls path continuity rather than a specific shape, it is geometry-agnostic and applicable to many antenna topologies, arrays, and frequency ranges. These features make the concept especially suitable for compact, efficient, reconfigurable wireless systems such as IoT sensor nodes, battery-powered beacons and asset trackers, space-limited embedded radios, and small multi-band arrays for platforms such as CubeSats, where routing bias lines are undesirable and quiescent power must be minimal. Practical implementation of the light source requires repeatable illuminance and robust light isolation. Key challenges to this include ambient-light variability, precise LED–LDR alignment, optical leakage to nearby conductors, and thermal/aging effects of the LED/LDR. A compact green SMD LED with an isolated light-pipe might mitigate these issues. Future research should focus on measuring switching transients versus illuminance and temperature and assessing long-term stability and aging.

## Figures and Tables

**Table 1:** Comparison of reconfiguration mechanisms.

Technique	RF-Board and DC Bias Lines	Typical Control/ Drive	Switching Speed	Layout Impact (Compact)
PIN diode	Yes (RF chokes, capacitors)	0.8–1.5 V	ns–µs	High
Varactor	Yes (RF chokes, capacitors)	Up to ~20 V (reverse), leakage nA–µA	ns–µs	Moderate
RF-MEMS (electrostatic)	Yes (high voltage driver)	~20–80 V actuation	µs–ms	Moderate–High
Mechanical actuation	Yes (stepper motors)	~5 V	ms–s	Low–Moderate (bulk)
Smart materials (fluidic/graphene)	Often No (pumps/bias off-board)	Varies (pumps/heaters/bias)	µs–s (tech-dependent)	High (integration complexity)
Optical (photoconductive LDR)	No (no RF-board DC lines)	Optical illumination (intensity-controlled)	ms (device-dependent)	Low

**Table 2:** Calculated and simulated resonance frequencies.

Arc Segment	LDR State	Length *L* (mm)	Calculated f (GHz)	Simulated f (GHz)	
				Resonance f (GHz)	Band (GHz)
Combined arcs	ON	109.7	Mode (1): 1.68	1.70	1.52–2.05
			Mode (2): 3.36	3.70	3.45–4.13
			Mode (3): 5.04	5.20	5.02 -> 7.00
Short arc	OFF	27.0	6.83	5.80	5.13–6.80
Long arc		76.3	2.42	2.45	2.14–2.80

## Data Availability

The original contributions presented in this study are included in the article. Further inquiries can be directed to the corresponding author.
